# Targeted next-generation sequencing analysis of pathogens and microecology in pediatric lower respiratory tract infections identifies risk factors for severe community-acquired pneumonia

**DOI:** 10.3389/fcimb.2026.1796357

**Published:** 2026-04-16

**Authors:** Hongquan Pi, Huaming Lin, Jiancheng Zhou, Huifang Liu, Shulan Liang, Runmiao Zhu, Dehua Li, Xiaocheng Lu, Meng Yang, Hanning Chen, Yuchuan Li

**Affiliations:** 1 Department of Cinical Laboratory, The Sixth Affiliated Hospital of Jinan University (Dongguan Eastern Central Hospital), Dongguan, Guangdong, China; 2 Guangzhou DaAn Clinical Laboratory Center Co. Ltd., YunKang Group, Guangzhou, China

**Keywords:** microbiome, Mycoplasma pneumoniae, pediatric lower respiratory tract infections, severe community-acquired pneumonia, targeted next-generation sequencing, throat swab

## Abstract

**Background:**

Traditional diagnostic methods have inherent limitations in the comprehensive assessment of the etiological spectrum and microecological characteristics of pediatric lower respiratory tract infections (LRTIs), particularly community-acquired pneumonia (CAP). Against this backdrop, the present study seeks to delineate the pathogen profile of children with LRTIs via targeted next-generation sequencing (tNGS), and further explore the associations between clinical manifestations, upper respiratory microbiome signatures and disease severity in pediatric CAP cases.

**Methods:**

A retrospective, single-center study was conducted on 2299 children with suspected lower respiratory tract infections. Throat swab samples from all patients underwent tNGS for pathogen detection. For 1845 CAP patients (293 SCAP, 1552 non-severe CAP [nsCAP]), clinical data and tNGS results were analyzed. Statistical comparisons, correlation analyses, and multivariate logistic regression were performed to identify factors associated with SCAP. Microbial diversity (Shannon/Simpson indices) and relative abundance of detected species were also analyzed.

**Results:**

Mycoplasma pneumoniae was the dominant atypical pathogen, with an outbreak peaking in July 2024. *M. pneumoniae* detection rate (35.8% vs. 8.9%, P<0.001) and relative abundance (RA) were significantly higher in SCAP than nsCAP patients and correlated positively with severity markers. Multivariate analysis identified *M. pneumoniae* positivity, older age, female sex, circulatory and metabolic diseases as independent risk factors for SCAP. In *M. pneumoniae*-negative patients, pathogens like *Streptococcus pneumoniae* and *Haemophilus influenzae* were more common in nsCAP. Upper respiratory microbial diversity was lower in SCAP patients. Increased RA of specific commensals like *Schaalia odontolytica* was a protective factor, while increased abundance of *Stenotrophomonas maltophilia* was a risk factor for SCAP. Compared to bronchoalveolar lavage fluid (BALF), throat swab tNGS showed high agreement for *M. pneumoniae* but higher detection of potential colonizers like *H. influenzae*.

**Conclusions:**

During the study period, *M. pneumoniae* was a key driver of SCAP in children. Beyond single-pathogen detection, decreased upper respiratory microbial diversity and shifts in colonizing bacteria abundances were associated with pneumonia severity, offering a new ecological perspective. Throat swab tNGS is valuable for pathogen screening. The associations between upper respiratory microbial features and severity highlight a potential ecological dimension of pneumonia pathogenesis.

## Introduction

Lower respiratory tract infections (LRTIs) represent a formidable global health challenge, particularly in pediatric populations where they remain a leading cause of hospitalization and mortality (Rodrigues and Groves, 2018; Wang et al., 2021). Community-acquired pneumonia (CAP), as the most severe clinical manifestation of LRTIs, demonstrates complex pathogen diversity encompassing bacterial, viral, fungal, and atypical pathogens, with increasing recognition of polymicrobial infections complicating clinical management (Gao et al., 2020; Liu et al., 2023). Traditional diagnostic methodologies, including microbial culture, serological testing, and targeted PCR, face significant limitations in clinical practice (Li et al., 2019; Huang et al., 2020; Gaston et al., 2022). Targeted next-generation sequencing (tNGS) serves as a practical high-throughput detection approach for common pathogens. By enriching predetermined pathogen targets through multiplex PCR or probe capture technology, it achieves a detection sensitivity of 84.6–96.4% for respiratory pathogens, which is significantly higher than the 40.7–57.3% sensitivity of traditional methods (Chen et al., 2024; Shang et al., 2025).

The diagnostic paradigm for pediatric LRTIs requires careful consideration of sampling methodologies. Bronchoalveolar lavage fluid (BALF) represents the gold standard for lower respiratory tract infections by directly sampling the site of infection, but its invasive nature limits routine use in children (Zhang et al., 2024a; Lian et al., 2025). In contrast, oropharyngeal (throat) swabs offer distinct advantages including non-invasiveness, simplicity, facilitating widespread clinical implementation (Serigstad et al., 2023). However, the oropharynx is a reservoir for both potential pathogens and commensal flora, making the interpretation of positive tNGS results challenging. The inherent ambiguity in distinguishing between causative pathogens and incidental upper airway colonization is a critical analytical challenge in respiratory microbiology. The inherent ambiguity in distinguishing between causative pathogens and incidental upper airway colonization is a critical analytical challenge in respiratory microbiology. While studies report good concordance for some pathogens between matched throat swab and BALF samples (Lian et al., 2024), the clinical significance of detecting common colonizers in throat samples remains poorly defined.

Concurrently, the analytical approach to tNGS data is evolving. Traditional tNGS reports have typically focused on the “qualitative” detection of pathogens, relying on thresholds such as Reads Per Million (RPM) to distinguish pathogens from background noise (Shang et al., 2025). Emerging evidence suggests that the relative abundance (RA), may better reflect microbial load and etiologic significance (Chen et al., 2023; Yin et al., 2024). Nevertheless, most tNGS panels and analyses are limited to predefined pathogenns, systematically excluding the broader community of upper respiratory commensals. This omission restricts a holistic assessment of the airway microbial milieu and its potential ecological imbalance, which could be informative for disease pathogenesis and severity (Chiu and Miller, 2019).

Therefore, to bridge these gaps, we aimed to evaluate whether comprehensive microbial features in throat swabs correlate with CAP severity. Severe CAP (SCAP) was defined per the 2011 Pediatric Infectious Diseases Society/Infectious Diseases Society of America (PIDS/IDSA) consensus guidelines, requiring the presence of either one major criterion (invasive mechanical ventilation, fluid-refractory shock, acute need for noninvasive positive pressure ventilation, or hypoxemia) or at least two minor criteria (Bradley et al., 2011). Non-severe CAP (nsCAP) included all other CAP cases. The central aim of this study is twofold: first, to evaluate the real-world pathogen landscape and the diagnostic performance of throat swab tNGS; second, and more importantly, to investigate whether specific microbial features in the oropharynx (including pathogen presence, relative abundance of commensals, and within-panel community diversity) are associated with the severity of CAP. We hypothesize that even a targeted snapshot of the upper respiratory microbial community holds valuable correlative information for disease stratification. The findings from this study are expected to provide not only etiological insights but also a novel, ecology-informed perspective for understanding the microbial correlates of disease severity in pediatric pneumonia.

We present a retrospective analysis of 2,299 children with suspected LRTIs, all providing admission throat swabs. From this cohort, we focused on 1,845 CAP patients, classifying them as SCAP (n=293) or nsCAP (n=1552) per the above criteria. Our analysis proceeded in three steps: (1) characterizing the pathogen spectrum; (2) identifying pathogen detection patterns and clinical factors associated with SCAP; and (3) investigating associations between SCAP and advanced microbial features, including relative abundance and within-panel diversity indices. A subset of 64 patients with matched BALF was analyzed to contextualize throat swab findings.

## Materials and methods

### Study design and sample collection

This retrospective study enrolled pediatric inpatients diagnosed with LRTIs at Dongguan Eastern Central Hospital between February 2024 and July 2025. The inclusion criteria were: (i) clinical and/or imaging evidence of LRTIs (pneumonia, tracheitis, bronchitis); (ii) age between 1 month and 14 years; and (iii) availability of throat swab specimens. Exclusion criteria comprised: (i) absence of tNGS testing; (ii) non-LRTIs or non-infections, (iii) incomplete clinical data. For patients with multiple hospitalization records during the study period, only included their first hospitalization record and related samples that met the study criteria. Among all the patients included in the study, a subset of which met the criteria for SCAP mention above.

Throat swab samples were collected within 24 hours of admission, before initiating any empirical systemic antibiotic treatment. Two swabs were collected from the posterior pharyngeal wall (at least three times per site) by trained physicians and immediately transferred into sterile tubes containing 0.5 mL of nucleic acid preservation solution. For patients whose swab results failed to fully explain the clinical presentation or whose condition deteriorated rapidly, 5 mL of BALF was collected via bronchoscopy and subjected to tNGS testing.

The study was approved by the Ethics Committee of Dongguan Eastern Central Hospital (Approval No.: MEC-SL-2025-075). As a retrospective analysis using anonymized patient records without additional interventions or sample collection, the institutional ethics committee granted a waiver of informed consent for the use of these data. The data collection protocol adhered to the principles of the Declaration of Helsinki.

### Clinical and microbiological data collection

The clinical and microbiological data of inpatients were obtained from the hospital’s clinical information system. These data include symptoms, underlying conditions, white blood cell count (WBC), neutrophil percentage, C-reactive protein (CRP), procalcitonin (PCT), other biochemical indicators, length of hospital stay, and information on antibiotic treatment. For each patient, the laboratory test obtained at admission (within 24 hours of hospitalization) were used as the baseline. We systematically collected antibiotic usage records after patient admission (post-sampling). However, detailed antibiotic treatment histories from the community or other healthcare facilities prior to hospitalization were incomplete or missing in most medical records.

### Targeted next-generation sequencing testing

For throat swab samples, the preservation tube containing the swab is vortexed, and then 800 µL is pipetted for nucleic acid extraction. For bronchoalveolar lavage fluid (BALF) samples, 800 µL is taken directly for nucleic acid extraction. Nucleic acid extraction involves total nucleic acid extraction (including DNA and RNA) using a nucleic acid extraction kit (Guangzhou Dayuanqi Biotechnology Co., Ltd.) in combination with the Smart32 automated nucleic acid extraction system (Guangzhou Da’an Gene Co., Ltd.), employing magnetic bead adsorption for automated extraction.

Total nucleic acid is used for cDNA synthesis with an RNA Reverse transcription kit (Guangzhou Da’an Gene Co., Ltd.). After quantification of cDNA/DNA using Qubit 4.0, 1 ng to 100 ng of the sample is taken for tNGS library construction using a multiplex library preparation kit (Guangzhou Dayuanqi Biotechnology Co., Ltd.), which involves adding a multiplex PCR amplification primer system targeting 158 respiratory pathogens and 28 colonizing bacteria of oral and upper respiratory tract (**Supplementary Tables 1, 2**). The amplified libraries are quantified using Qubit 4.0, followed by pooling of multiple libraries. With each batch of libraries, both negative and positive controls were included and processed. DNB preparation is performed using the DNBSEQ DNB Preparation Kit (MGI Tech Co., Ltd., Shenzhen). Sequencing is carried out using the DNBSEQ-G99 SM FCL SE100/PE50 sequencing reagent (MGI Tech Co., Ltd., Shenzhen) on the DNBSEQ-G99 sequencer (MGI Tech Co., Ltd., Shenzhen), employing a single-end 50 bp (SE50) sequencing strategy with an average total data volume of 0.4M reads per sample.

Low quality reads were removed with fastp (v0.21.0), and then the clean data was aligned to a custom, in-house reference database with the burrows-wheeler aligner (BWA) (version 0.7.17-r1198). The reference database was constructed specifically for this tNGS panel and contains the genomic sequences of all 186 targeted organisms (158 pathogens and 28 colonizing bacteria). The sequences were curated from authoritative public repositories (e.g., NCBI RefSeq) and verified for specificity. For candidate pathogens identified through tNGS analysis, a rigorous filtration and verification process was implemented. First, species identification was filtered based on a 95% sequence identity threshold to retain only species and sequences with reliable alignment. Second, reagent/engineered microorganisms and laboratory background contaminants were excluded. The laboratory maintains a reagent/engineering microorganism database updated per run and a laboratory background microorganism database updated weekly based on actual conditions. To eliminate inter-sample sequencing data variation and standardize reporting thresholds, the read count for each sample were normalized to 0.4 million per sample. Credible species were determined based on the following criteria: (i) Nomalized Read count aligned to the species (≥10 reads); (ii) Number of distinct amplicons detected for the species (≥2 amplicons); (iii) Ratio of reads for the microorganism in the sample compared to the negative control (≥20); Finally, the report was generated by integrating microbial characteristics, patient history, clinical diagnosis, and other laboratory test results.

### Multivariable logistic regression analysis

To investigate the association between microbial detection and disease severity (severe vs. non-severe), we employed multivariable logistic regression models to evaluate the independent effect of each microbial species while controlling for key clinical confounders. First, based on tNGS data, we screened for microbial species that were detected with sufficient frequency across the samples. Disease severity (condition) was defined as a binary variable (severe = 1, non-severe = 0). To control for potential confounding, the following clinical variables were included: sex, metabolic system disorders, circulatory system disorders, fever, cough, vomiting, nervous system disorders, abnormal liver function tests, metabolic acidosis, season, and month of age. Due to the widespread lack of antibiotic use history prior to admission, it was not included as a covariate in the model. The construction and validation of the Multivariable Logistic Regression Analysis are detailed in the **Supplementary Methods**. For each microbial species, a multivariable logistic regression model was constructed as follows:


$$\begin{array}{c} \text{logit}(\text{P}(\text{condition }=\;1))\\ \;=\;{\text{β}}_{0}\;+\;{\text{β}}_{1}\;\times \;(\text{Detection}\;\text{Status})\\ \;+\;\sum {\text{β}}_{\text{i}}\;\times \;(\text{Clinical}\;{\text{Confounder}}_{\text{i}}) \end{array}$$

where β_0_ is the intercept, β_1_ is the regression coefficient for the microorganism, and β_i_ are the regression coefficients for the clinical confounders. The models were fitted using the maximum likelihood estimation method. From each fitted model, the regression coefficient and its standard error were extracted for the microorganism, and the odds ratio (OR = exp(β_1_)) along with the 95% confidence interval (CI) were calculated. Statistical significance was assessed using the Wald test, yielding the corresponding p-value. All statistical analyses were performed in R (version 4.4.1) using the glmfunction for model fitting. Confidence intervals were computed based on the asymptotic normality of the model coefficients.

To analyze the association between the relative abundance (RA) of different microorganisms and disease severity, we also constructed the following multivariable logistic regression model:


$$\begin{array}{c} \text{logit}(\text{P}(\text{condition }=\;1))\\ \;=\;{\text{β}}_{0}\;+\;{\text{β}}_{1}\;\times \;(\text{RA}\%)\;+\;\sum {\text{β}}_{\text{i}}\;\times \;(\text{Clinical}\;{\text{Confounder}}_{\text{i}}) \end{array}$$

Here, RA% represents the corrected read proportion of each bacteria relative to all bacteria in the sample, indicating its relative microbial abundance. It is calculated as: RA% = (Corrected read count for the species/Total reads for all species in the sample) × 100%. The read counts were corrected based on the number of target amplicons for that species to mitigate bias from primer amplification efficiency. It should be noted that in targeted sequencing based on multiplex PCR, the sequence read counts of different species are influenced by primer binding efficiency, amplification bias, and genomic characteristics (such as copy number). Therefore, the calculated RA reflects the proportional representation of each target species within the sequencing library. Although, after normalizing for amplicon quantities, RA can be used to compare the relative strengths of different species within the same sample or the relative changes of the same species across different samples, it cannot be directly equated to the absolute quantity or abundance of microorganisms in the sample unless validated in parallel with quantitative methods (e.g., qPCR).

### Random Forest modeling and feature importance assessment

To systematically assess the incremental predictive value of each microbial species in the respiratory targeted microbial community for severe pneumonia, we developed a species-level incremental contribution analysis method based on Random Forest, building on a clinical prediction model. This method quantifies the improvement in predictive performance after incorporating each microbial species into the clinical model, thereby identifying microorganisms that provide significant incremental value for distinguishing between severe and non−severe cases.

First, we constructed a baseline clinical prediction model using the Random Forest algorithm. In each iteration, stratified sampling was employed to randomly partition the data into a training set (80%) and a test set (20%). The model parameters were set as follows: ntree= 1000, and the default mtryparameter (square root of the number of features) was used. The area under the receiver operating characteristic curve (AUC) was calculated on the test set as the model performance metric. This process was repeated 10 times to obtain a stable performance estimate. Subsequently, building on the baseline clinical model, we evaluated the incremental predictive value of each microbial species individually. For the i-th species, its abundance data were combined with the clinical features to construct a new hybrid model. By comparing the difference in AUC between the hybrid model and the baseline model on the same test set, the AUC increment (ΔAUC) contributed by that species was computed. All analyses were conducted in the R environment. Key R packages used included: randomForest​ (for Random Forest modeling), pROC​ (for ROC curve analysis), caret​ (for data splitting), and doParallel​ (for parallel computing). Code implementation ensured reproducibility by setting a fixed random seed to control stochastic processes in data partitioning and model training.

### Statistical analyses

All statistical analyses were performed using R software (version 4.4.1). A p-value < 0.05 was considered statistically significant. Continuous variables were first tested for normality. Data conforming to a normal distribution are presented as mean ± standard deviation, while data not conforming to a normal distribution are presented as median (interquartile range, IQR). Comparisons between two groups were conducted using the t-test or the Wilcoxon rank-sum test; comparisons among multiple groups were performed using ANOVA or the Kruskal-Wallis test (ANOVA was used if homogeneity of variance was met, otherwise the Kruskal-Wallis test was used). Categorical variables are expressed as number of cases (percentage), and comparisons between groups were made using the chi-square test, Fisher’s exact test, or the Wilcoxon rank-sum test. The “cor.test” function from the stats package in R was used to assess the Spearman correlation between clinical features and species detection. Graphical visualization was completed using the ‘ggplot2’ package in R, and the colors and layout were subsequently refined using Adobe Illustrator(version 25.4.1).

## Results

### Clinical characteristics of patients

**Figure 1:**
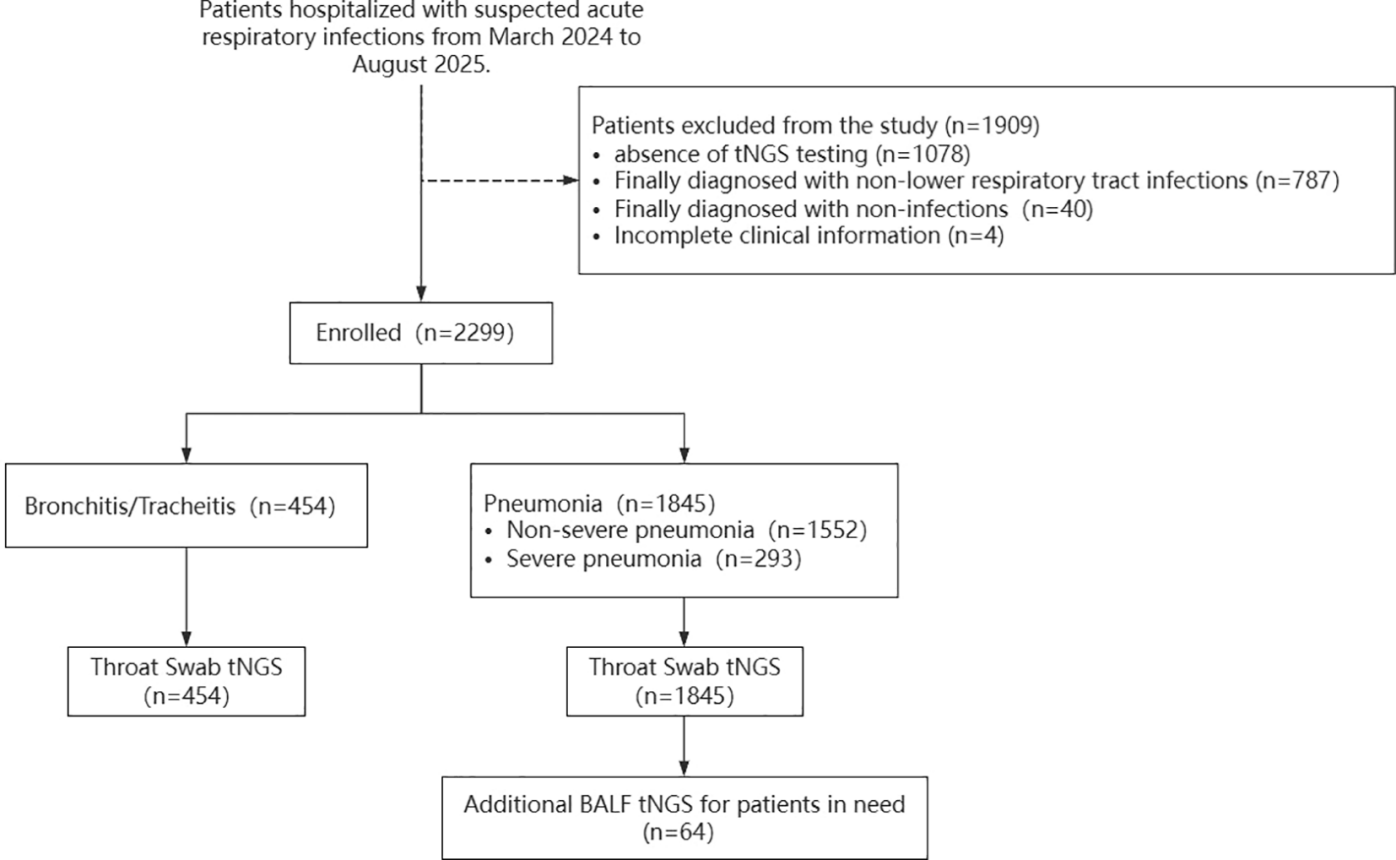
Participant recruitment for the study.

A total of 2299 children with suspected LRTIs were enrolled in this study, all of whom underwent throat swab tNGS testing. Among these, 1,845 were diagnosed with pneumonia (including 293 cases of severe pneumonia), and 454 with tracheitis/bronchitis (**Figure 1**). Among all 2299 patients, differences in age, cough, digestive disorders, autoimmune diseases, nervous system diseases, urinary system diseases, and length of hospital stay were observed between pneumonia and tracheitis/bronchitis (**Supplementary Table 3**).

### Distribution characteristics of pathogens detected by tNGS

**Figure 2:**
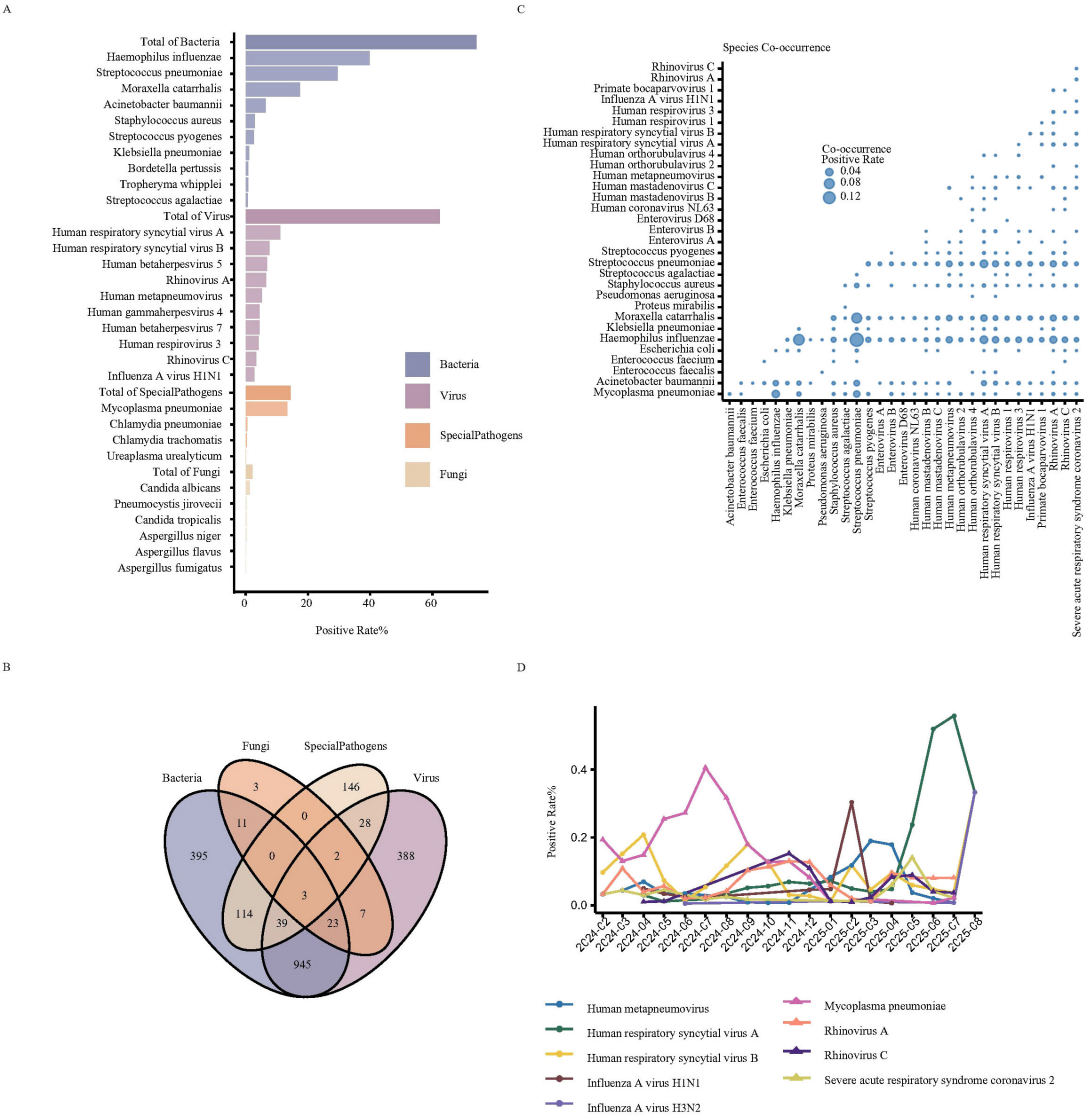
Pathogenic spectrum identified through tNGS among 2299 patients. **(A)** Detection rates of bacteria, viruses, atypical pathogens, and fungi in throat swab samples from 2299 patients (only the top 10 species with the highest detection rates for bacteria and viruses are shown; complete results are shown in **Supplementary Figure S1**). And all the other figures, tables, and captions are correct, and that all figures are of the highest quality/resolution. **(B)** Co-detection distribution of different pathogen types. **(C)** Co-detection patterns among 32 clinically significant pathogens. **(D)** Epidemic trends of *M. pneumoniae*, *B. pertussis*, and 20 respiratory viruses during the study period (species with a difference in detection rates of ≥10% between the highest and lowest months are presented, complete results are shown in Figure S).

Throat swab samples from all 2,299 children were tested by tNGS. Results were positive in 2103 cases (91.47%), with 67 distinct pathogens detected. These included 23 bacteria, 34 viruses (26 RNA viruses, 8 DNA viruses), 6 fungi, and 4 atypical pathogens (e.g., *Mycoplasma*or *Chlamydia*). The detection rates for bacteria, viruses, fungi, and atypical pathogens were 74.16%, 62.42%, 2.13%, and 14.44%, respectively. The top five bacteria detected were *Haemophilus influenzae*, *Streptococcus pneumoniae*, *Moraxella catarrhalis*, *Acinetobacter baumannii*, and *Streptococcus pyogenes*. The top five viruses were Respiratory syncytial virus A (RSV-A), Human herpesvirus 5 (Cytomegalovirus, CMV), Respiratory syncytial virus B (RSV-B), Rhinovirus A, and Human metapneumovirus (HMPV). Among atypical pathogens, *M. pneumoniae* was predominant (**Figure 2A**; **Supplementary Figure 1**). Among all positive samples, mixed detections involving multiple pathogen types were common, primarily co-detections of bacteria-virus, bacteria-atypical pathogen, and virus-atypical pathogen (**Figure 2B**). High co-detection rates were observed among *H. influenzae*, *S. pneumoniae*, and *M. catarrhalis*. Co-detections involving *M. pneumoniae* with RSV, Bocavirus, Rhinovirus, HMPV, Enterovirus, as well as viral co-detections, were also present in notable proportions (**Figure 2C**).

We analyzed the epidemic trends of 32 key respiratory viruses and atypical pathogens from February 2024 to August 2025. An outbreak of *M. pneumoniae* presence was observed from February to November 2024, peaking in July 2024 with a detection rate as high as 41.82%, after which it declined to 1.06% by January 2025. Notably, following the *M. pneumoniae* epidemic, Influenza A virus, HMPV, SARS-CoV-2, and RSV became the dominant pathogens in children with LRTIs in February, March-April, May, and June-August 2025, respectively, showing distinct seasonal succession characteristics (**Figure 2D**; **Supplementary Figure 1**).

Analysis by age group revealed that both the overall pathogen positive rate and viral positive rate were significantly higher in the infant (0–1 years) and toddler (1–3 years) group than in preschool (3–6 years) and school-age (6–14 years) children (**Supplementary Figure 2**). Conversely, the positive rate for atypical pathogens (mainly *M. pneumoniae*) was higher in preschool and school-age children. Viral co-detections were more common in infants, toddlers, and preschoolers but markedly decreased in school-age children (**Supplementary Figure 3**).

### Comparative pathogen detection in BALF versus throat swab

**Figure 3:**
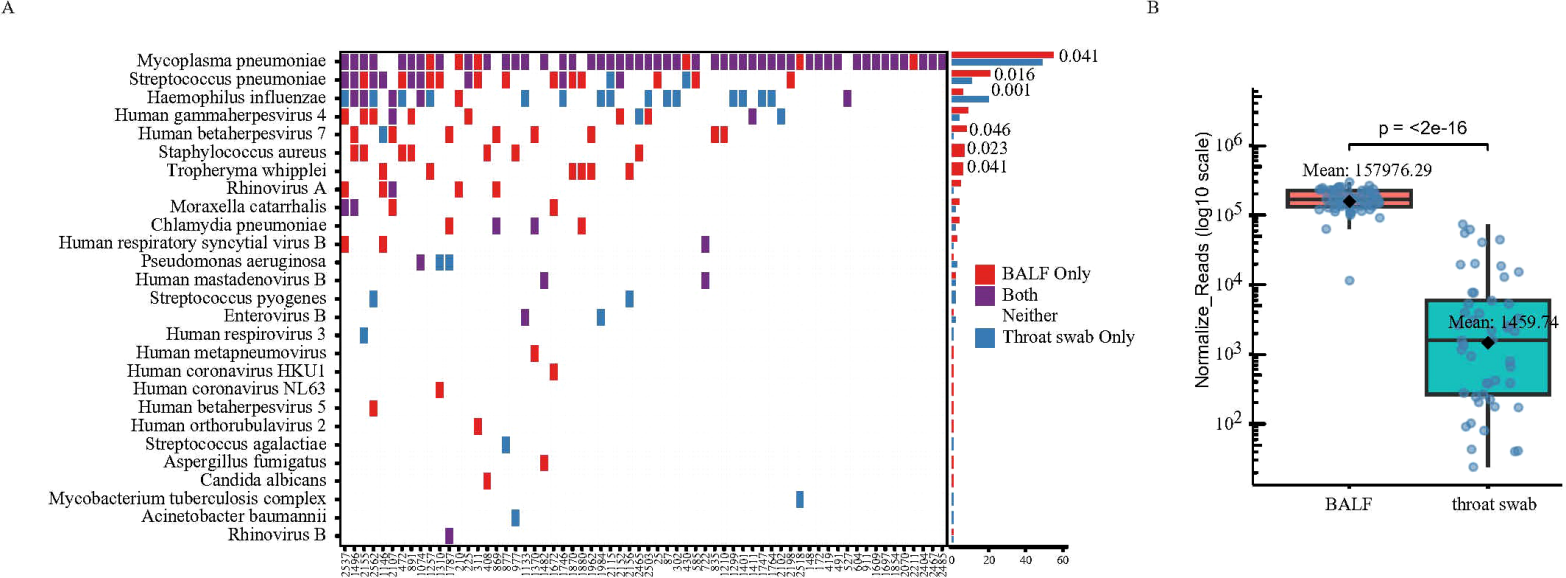
Differences in the detection of pathogens and microbial diversity in BALF versus throat swab **(A)** Comparison of pathogen detection between throat swab and BALF samples in 64 children who underwent both tests. **(B)** Comparison of *M. pneumoniae* normalized read counts between throat swab and BALF samples from the 64 children.

To understand the detection patterns of tNGS in the lower versus upper respiratory tract in a clinical setting, we analyzed a subset of pneumonia patients who underwent both throat swab and BALF testing. It is important to note that this subset is not representative of the general CAP population: BALF was collected based on clinical need (e.g., diagnostic uncertainty or disease progression), resulting in a cohort heavily skewed toward severe cases (61 SCAP, 3 nsCAP). BALF samples were obtained an average of 2.8 days after the initial throat swab. Therefore, the following comparisons aim to describe the pathogen profiles and concordance between these two anatomically distinct sites in a diagnostically challenging cohort, rather than to establish their equivalence or to validate throat swab as a direct surrogate for BALF. A total of 64 patients (61 SCAP, 3 nsCAP) were included. *M. pneumoniae* detection accounted for 85.93% (55/64) of all samples, with a 100% detection rate in BALF, while tNGS detected 89.09% (49/55) of these (**Figure 3A**). The normalized read count of *M. pneumoniae* in BALF was significantly higher than in throat swab samples (**Figure 3B**). Notably, *H. influenzae* was detected significantly more frequently in throat swab samples than in BALF (n=20 vs. n=6, p-value=0.001), suggesting that *H. influenzae* detected in throat swabs may primarily represent upper respiratory tract colonization. Furthermore, we found that the detection rates of *S. pneumoniae* (n=11 vs. n=20, p-value=0.016), *Staphylococcus aureus* (n=0 vs. n=7, p-value=0.023), *Tropheryma whipplei* (n=0 vs. n=6, p-value=0.041), and human herpesvirus 7(n=1 vs. n=8, p-value=0.046) were significantly higher in BALF compared to throat swab samples.

### Pathogen detection and targeted microbial features in throat swabs associated with SCAP

We focused on analyzing the tNGS pathogen detection results in 1845 children with CAP. Among the pneumonia patients, 293 were SCAP, while the remaining 1552 nsCAP (**Figure 1**). SCAP patients were older, primarily manifested as a significantly higher proportion in the school-age (6–14 years) group compared to nsCAP patients (**Supplementary Table 4**). Furthermore, SCAP patients had longer hospital stays, a higher prevalence of circulatory system diseases, and a higher neutrophil percentage than nsCAP patients.

**Figure 4:**
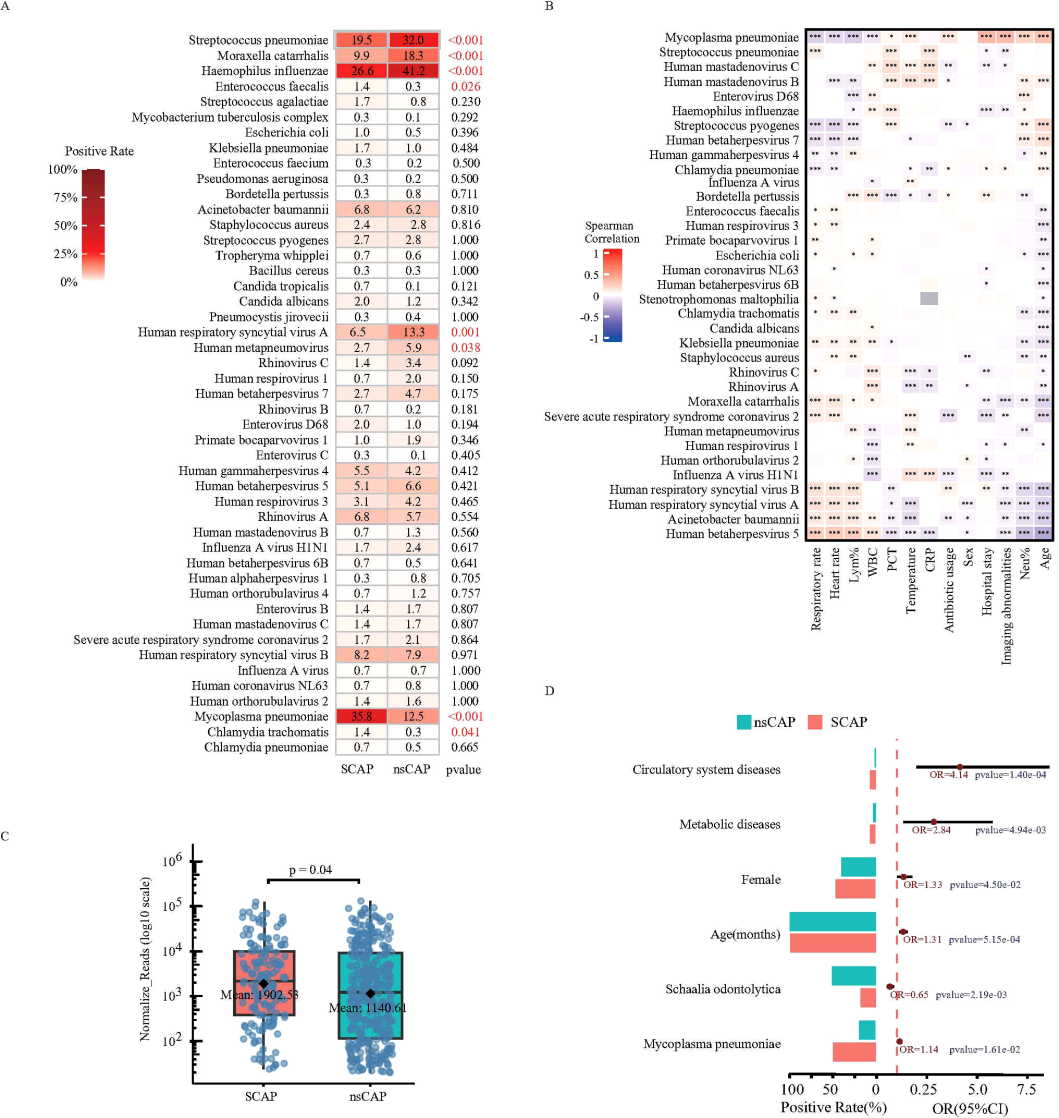
Pathogen distribution and infection patterns in SCAP versus nsCAP patients. **(A)** Differences in pathogen detection rates between SCAP and nsCAP patients (only pathogens with detection rates ≥0.1% are shown, complete results are shown in **Supplementary Figure 7**). **(B)** Spearman correlation analysis between the detection of 25 clinically significant pathogens and clinical features. **(C)** Difference in *M. pneumoniae* normalized read counts between SCAP and nsCAP patients. **(D)** Multivariable-adjusted logistic regression analysis of SCAP versus nsCAP patients (only factors with p-value <0.05 are shown).

The detection rate of *M. pneumoniae* in throat swabs (35.8% vs. 8.9%, P < 0.001) and its average sequencing normalized read count (1902.53 vs. 1149.10, P = 0.043) were both significantly higher in SCAP patients than in nsCAP patients (**Figures 4A, C**). The detection rates of *S. pneumoniae* (SACP = 19.5% vs nsCAP=2.0%, p-value<0.001), *H. influenzae* (26.6% vs 41.2%, p-value<0.001), *M. catarrhalis*(9.9% vs 18.3%, p-value<0.001), RSV-A(6.5% vs 13.3%, p-value<0.001), and Human metapneumovirus (2.7% vs 5.9%, p-value=.038)were significantly higher in nsCAP than in SCAP (**Figure 4A**; **Supplementary Figure 7**). However, further subgroup analysis of *M. pneumoniae*-positive and *M. pneumoniae*-negative patients revealed that the differences in detection rates for *S. pneumoniae*, *H. influenzae*, and *M. catarrhalis* were only observed in *M. pneumoniae*-negative patients (**Supplementary Figure 4**). Furthermore, within *M. pneumoniae*-positive patients, the detection rates of *Streptococcus agalactiae* and *S. aureus* were higher in SCAP than nsCAP, although the differences were not statistically significant.

Spearman correlation analysis between pathogens (including other upper respiratory colonizers) and clinical features showed that *M. pneumoniae* detection was significantly positively correlated with length of hospital stay, neutrophil percentage, age, and imaging abnormalities, and significantly negatively correlated with lymphocyte percentage (all P < 0.01) (**Figure 4B**), further confirming the association of *M. pneumoniae detection* with disease severity. Conversely, *S. pneumoniae*, *H. influenzae*, and *M. catarrhalis detection* showed negative correlations with length of stay, imaging abnormalities, and antibiotic use, consistent with their enrichment in nsCAP patients. Notably, *Haemophilus parainfluenzae*, *Veillonella parvula*, *Schaalia odontolytica*, *Streptococcus mitis*, and *Actinomyces naeslundii* were significantly negatively correlated with length of stay, imaging abnormalities, and antibiotic use, suggesting a need for greater attention to the relationship between upper respiratory colonizers and clinical symptoms.

Multivariate logistic regression analysis was performed to assess the independent effects of clinical factors and each microorganism on the risk of severe pneumonia. Results showed that circulatory system diseases (OR = 4.14, 95% CI: 1.97-8.59), metabolic system diseases (OR = 2.84, 95% CI: 1.33-5.76), female sex (OR = 1.33, 95% CI: 1.00-1.77), increasing age (OR = 1.31, 95% CI: 1.12-1.52), and positive detection of *M. pneumoniae* (OR = 1.13, 95% CI: 1.02-1.26) were independent risk factors for SCAP (**Figure 4D**). Notably, detection of *S. odontolytica* was a protective factor against SCAP (OR = 0.65, 95% CI: 0.48-0.84). Analysis of *M. pneumoniae*-negative patients (186 SCAP, 1345 nsCAP) revealed that the aforementioned clinical factors and microbial detection remained significantly associated with SCAP (**Supplementary Figure 5A**).

### SCAP risk assessment based on microbial relative abundance

**Figure 5:**
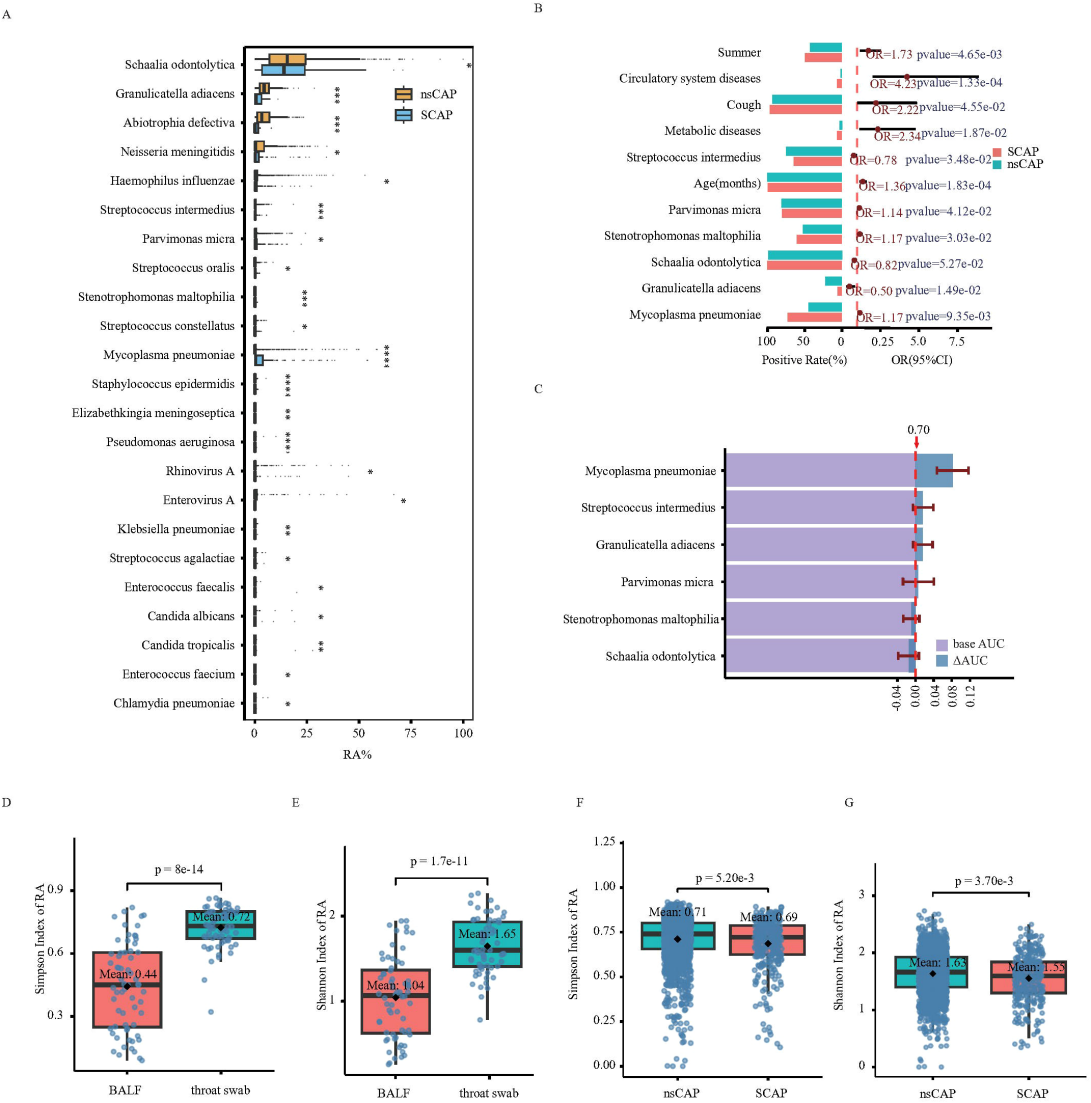
Analysis of microbial diversity between SCAP and nsCAP patient groups. **(A)** Differences in relative abundance (RA) of various pathogens between SCAP and nsCAP patients (only species with p-value <0.05 are shown). **(B)** Multivariable-adjusted logistic regression analysis of SCAP versus nsCAP patients based on RA (only factors with p-value <0.05 are shown). **(C)** ΔAUC values for different species in discriminating between SCAP and nsCAP. **(D)** Distribution of the Simpson index in pair throat swab and BALF samples. **(E)** Distribution of the Shannon index in pair throat swab and BALF samples. **(F)** Distribution of the Simpson index in SCAP and nsCAP patients. **(G)** Distribution of the Shannon index in SCAP and nsCAP patients.

To explore the significance of upper respiratory tract colonizers in assessing SCAP risk, we also analyzed the association between relative microbial abundance and SCAP. Results showed that the RA of *Abiotrophia defectiva* (mean RA: nsCAP 5.07% vs. SCAP 2.18%, p-value=0.0002), *Streptococcus intermedius* (mean RA: nsCAP 0.27% vs. SCAP 0.40%, p-value=0.0003), *Granulicatella adiacens* (mean RA: nsCAP 1.66% vs. SCAP 5.02%, p-value=0.0005), and *S. odontolytica* (mean RA: nsCAP 15.38% vs. SCAP 17.12%, p-value=0.01) were significantly higher in nsCAP patient samples compared to SCAP (**Figure 5A**), while the RA of *M. pneumoniae* was significantly lower in nsCAP (mean RA: nsCAP 2.45% vs. SCAP 4.46%, p-value<0.0001), consistent with the normalized read count difference(mean normalized read count: nsCAP 1140.61 vs. SCAP 1902.53, p-value=0.04) (**Figure 4C**).

Multivariate logistic regression analysis incorporating RA showed that increased RA of *M. pneumoniae* (OR = 1.14, 95% CI: 1.01-1.30), *Stenotrophomonas maltophilia* (OR = 1.15, 95% CI: 1.02-1.30), and *Parvimonas micra* (OR = 1.14, 95% CI: 1.01-1.30) positively correlated with SCAP. In contrast, increased RA of *S. odontolytica* (OR = 0.82, 95% CI: 0.67-1.00), *S. intermedius* (OR = 0.77, 95% CI: 0.61-0.98), and *G. adiacens* (OR = 0.50, 95% CI: 0.29-0.87) negatively correlated with SCAP (**Figure 5B**).

To quantify how changes in the RA of specific microorganisms improve the prediction of SCAP, we performed a feature importance analysis based on the Random Forest algorithm, as detailed in the Methods. The baseline clinical model achieved an AUC of 0.70 (95% CI: 0.63 to 0.77) (**Figure 5C**). The inclusion of *M. pneumoniae* provided the most substantial improvement, elevating the AUC to 0.78 (95% CI: 0.76 to 0.83), corresponding to a ΔAUC of 0.08,while other microbial features provided minimal incremental value. In the same subgroup of *M. pneumoniae*-negative patients, the RA of these microbes also remained significantly associated with SCAP (**Supplementary Figure 5B**). The full performance metrics of the baseline and the top-performing hybrid models, including sensitivity and specificity at the optimal cut-off point, are provided in **Supplementary Table 5**.

Beyond changes in single species abundance, we assessed microbial diversity and evenness using the Simpson and Shannon indices. Analysis of 64 paired throat swab and BALF samples showed that microbial diversity in throat swabs was significantly higher than in BALF samples (**Figures 5D, E**), suggesting that the Simpson and Shannon indices calculated from 186 species in the tNGS data could partially reflect the diversity of species in the upper and lower respiratory tracts. Consequently, we analyzed the upper respiratory microbial community diversity in SCAP and nsCAP children. The results showed that both the Shannon and Simpson indices were significantly lower in SCAP children than in nsCAP children, indicating reduced species diversity and evenness in the panel. (**Figures 5F, G**). Subgroup analysis showed that in patients negative for *M. pneumoniae*, the Shannon and Simpson indices of SCAP patients were still significantly lower than those of nsCAP patients (**Supplementary Figure 6**).

## Discussion

Through systematic analysis of throat swab tNGS data from 2299 children with LRTIs, this study comprehensively delineated the pathogen spectrum characteristics of pediatric respiratory infections. More importantly, via in-depth analysis of the pneumonia subgroup, this study investigated the clinical risk factors, key pathogen features, and targeted upper respiratory microbial community changes associated with SCAP. The study found that during the 2024–2025 study period, the outbreak and infection of *M. pneumoniae* were the core microbial factors driving SCAP in children. Concurrently, underlying conditions like circulatory or metabolic system diseases also significantly increased the risk of severe illness. Furthermore, this study innovatively revealed, from a respiratory microbiome perspective, that decreased targeted upper respiratory microbial diversity and changes in the abundance of specific commensal bacteria were associated with disease severity, providing a new ecological perspective for assessing the risk of severe pneumonia. Our study identifies significant associations between microbial features detectable in readily obtainable throat swabs and the clinical outcome of severe pneumonia. It is crucial to emphasize that these associations do not necessarily indicate that the throat microbiota directly drives lower respiratory pathology; rather, they may reflect shared systemic host responses, ecological disturbances relevant to disease susceptibility, or the oropharyngeal presence of key pathogens.

This study was conducted during a regional outbreak of *M. pneumoniae* (Tong et al., 2022; Ma et al., 2024). Our data clearly showed that *M. pneumoniae* detection was highly enriched in SCAP children, with both its detection rate and normalized read counts (representing relative pathogen abundance) significantly higher than in nsCAP children. This suggests that high-abundance detection of *M. pneumoniae* in throat swabs is an important factor leading to severe pneumonia. Multivariate regression analysis confirmed that *M. pneumoniae* detection was an independent risk factor for SCAP and significantly positively correlated with severity indicators like prolonged hospital stay and imaging abnormalities. Feature importance analysis from the random forest model further confirmed that the RA of *M. pneumoniae* was the most important microbial factor distinguishing SCAP from nsCAP. These results collectively emphasize that, in the current context, *M. pneumoniae* is a key pathogen requiring focused attention in children with severe pneumonia. To address the potential confounding effect of the concurrent *M. pneumoniae* outbreak on the observed ecological associations, we performed a critical subgroup analysis restricted to *M. pneumoniae*-negative patients. Notably, even after complete exclusion of all *M. pneumoniae*-positive cases, the key microbial associations with pneumonia severity persisted. Specifically, within this subgroup, SCAP patients still demonstrated significantly lower alpha-diversity indices (Shannon and Simpson) of the targeted microbial community compared to nsCAP patients. Furthermore, the protective association linked to the detection and higher RA of the commensal *S. odontolytica*, as well as the risk association with a higher RA of *S. maltophilia*, remained statistically significant. These findings strongly suggest that the observed correlations between an altered upper respiratory microbial ecology and disease severity are not merely artifacts of the dominant pathogen outbreak but may reflect more fundamental and generalizable host-microbe interactions relevant to CAP pathogenesis.

Previous studies suggest that co-detection of *M. pneumoniae* with bacteria (e.g., *S. aureus*) may exacerbate illness, potentially through mechanisms involving intensified inflammatory responses (Dai et al., 2018). However, the picture may be more complex when considering throat swab samples. Results from this cohort suggested that within *M. pneumoniae*-positive pneumonia patients, the detection rates of *S. agalactiae* and *S. aureus* were higher in SCAP than nsCAP, though not significantly. This hints that co-detection of *M. pneumoniae* with *S. aureus* or *S. agalactiae* might worsen the infection. The lack of significant difference in their co-detection between SCAP and nsCAP may stem from the complexity of upper respiratory bacterial flora (Dong et al., 2025).

Simultaneously, among all pneumonia patients, the detection rates of *H. influenzae*, *S. pneumoniae*, and *M. catarrhalis* were significantly higher in nsCAP than SCAP. However, this difference was mainly evident in *M. pneumoniae*-negative patients (**Supplementary Figure 3**), with no significant difference observed in *M. pneumoniae*-positive patients. *H. influenzae*, *S. pneumoniae*, and *M. catarrhalis* primarily colonize the upper respiratory tract (Sulikowska et al., 2004; Langelier et al., 2018a; Shang et al., 2025). Their decreased detection in severe infection patients may reflect a reduction in upper respiratory microbiome diversity. Patients with LRTIs exhibit upregulation of pathways related to innate immune response, NF-κB signaling, cytokine production, and type I interferon response, affecting local airway immunity and consequently influencing microbiome replication or survival (Langelier et al., 2018a). In *M. pneumoniae*-positive patients, this diversity difference was not significant, we speculate that *M. pneumoniae*, as a strong pathogen, induces more severe systemic inflammation, potentially leading to a general reduction in the microbiome (Dai et al., 2018; Dong et al., 2025). This also implies that factors driving severity in *M. pneumoniae*-negative patients may differ significantly from those in *M. pneumoniae*-positive patients.

A breakthrough finding of this study lies in moving beyond single pathogen detection, preliminarily revealing a potential association between the upper respiratory microbial ecology, as analyzed by tNGS, and pneumonia severity. In non-targeted metagenomic (mNGS) analysis, relative abundance is often used to judge pathogens (Liu et al., 2022; Sun et al., 2022) and analyze microbiome diversity (Zhang et al., 2024b; Lian et al., 2025; Huang et al., 2025). Traditional tNGS reports typically focus on “qualitative” pathogen detection, relying on thresholds like RPM to distinguish pathogens from background noise. Recent studies have explored using quantitative/semi-quantitative metrics and RA to improve tNGS interpretation, indicating that tNGS RA can partially reflect microbial load (Yin et al., 2024; Shang et al., 2025). This study found that incorporating common oral anaerobes and other upper respiratory colonizers (e.g., *Staphylococcus*, *Streptococcus*, *Prevotella*, *V. parvula*) allows for assessment of the upper respiratory microbial composition and its ecological balance. Respiratory colonizers may play a “gatekeeper” role in maintaining respiratory health by competitively inhibiting pathogen colonization or modulating local immunity (Man et al., 2017; Diao et al., 2022). Multiple studies show reduced diversity in LRTIs in immunocompromised individuals, potentially serving as an ecological marker of infection (Sulikowska et al., 2004; Langelier et al., 2018a; Shang et al., 2025; Langelier et al., 2018b). We found that the species diversity (Shannon/Simpson indices) of the pharyngeal microbial community was significantly lower in SCAP patients than nsCAP patients, further suggesting that reduced diversity of certain common upper respiratory colonizers is associated with disease severity. The observed reduction in diversity in SCAP patients’ throat swabs could be either a predisposing factor for severe disease or a consequence of the systemic inflammatory state, a distinction our cross-sectional study cannot unravel.

However, it is important to clarify that the “diversity” and “microecological” analyses in this study are confined to the species within the targeted panel. This method is not an unbiased metagenomic (mNGS) analysis. Therefore, the diversity indices calculated based on the tNGS panel reflect the diversity within this targeted set of microorganisms, not the complete diversity of the respiratory microbial community. Furthermore, in tNGS detection based on multiplex PCR, the read counts for different species are also influenced by primer binding efficiency, amplification bias, and genomic characteristics (such as gene copy number). Nonetheless, as the majority of microorganisms included in the panel are common respiratory opportunistic pathogens or colonizers, and similar primer design standards were applied, this approach can, to some extent, reflect certain microecological features under a controlled study design. It serves as a simplified yet practical method for assessing respiratory microbial characteristics.

More in-depth multivariate regression analysis highlighted the independent predictive value of abundance changes in specific oral commensals. For instance, increased relative abundance of *S. odontolytica*, *A. defectiva, S. intermedius*, and *G. adiacens* were protective factors against SCAP. This aligns with the higher diversity of normal upper respiratory commensals in nsCAP patients. This association could be explained if such commensals exert a “biological barrier” effect by competing for nutrients, occupying ecological niches, producing antimicrobial substances, or modulating local immune responses, thereby inhibiting pathogen colonization and maintaining microecological stability (Man et al., 2017; Diao et al., 2022). However, an equally plausible alternative explanation is that the systemic inflammation and physiological stress of severe pneumonia itself, or preceding factors (e.g., antibiotic exposure), leads to a depletion of these commensals. Thus, the observed correlation may reflect either a protective role of the resident flora, a consequence of severe disease, or a combination of both. Besides *M. pneumoniae*, increased abundance of *S. maltophilia* was also a significant risk factor. Additionally, this study defined SCAP based on a composite endpoint. Future research could further explore the associations between the aforementioned microbial characteristics and specific severity components such as hypoxemia and pleural effusion using larger sample sizes, which would help identify microbial markers with greater predictive value for specific clinical phenotypes.

At the diagnostic technology level, this study evaluated the clinical utility of throat swab tNGS. Although throat swab tNGS showed a high positive percent agreement (89.09%) for detecting *M. pneumoniae* compared to the “gold standard” BALF samples, BALF samples demonstrated higher detection rates and pathogen abundance (read counts), consistent with prior studies (Deng et al., 2023; Yang et al., 2024). This affirms the effectiveness of throat swab tNGS as a non-invasive, convenient method for screening most respiratory pathogens (Serigstad et al., 2023; Yang et al., 2024; Zhang et al., 2024b; Lian et al., 2025). However, the significantly higher detection of *H. influenzae* in throat swabs versus BALF suggests it more likely represents upper respiratory colonization. While *S. pneumoniae*, *S. aureus*, and certain herpesviruses were enriched in BALF, previous studies indicate that lower respiratory opportunistic pathogens, including *S. pneumoniae* and *M. catarrhalis*, frequently colonize the upper respiratory tract (Sulikowska et al., 2004; Langelier et al., 2018a; Shang et al., 2025). Therefore, this underscores the importance of rationally interpreting tNGS results considering the sampling site and carefully distinguishing colonization from active infection, especially in severe, complex, or poorly responsive cases. BALF tNGS testing holds irreplaceable value in identifying true lower respiratory pathogens (Chen et al., 2024; Ding et al., 2025; Shang et al., 2025). Our study supports a nuanced application of throat swab tNGS in pediatric CAP. During a dominant outbreak (e.g., *M. pneumoniae)*, it serves as a rapid screening and severity triage tool. In non-severe cases, it can broaden the etiologic profile to guide therapy, though results for common colonizers require cautious interpretation. Bronchoscopy with BALF sampling should be strongly considered in the following scenarios, where throat swab findings are insufficient for definitive management: Immunocompromised patients with severe or atypical pneumonia, where the spectrum of potential pathogens is vast and colonization is less distinguishable. Severe or life-threatening CAP not responding to initial empirical therapy, where confirming the true lower respiratory pathogen is critical. When investigating non-infectious mimics.

This study has several limitations. First, as a single-center retrospective study, selection bias may exist, and the generalizability of conclusions needs validation in multi-center prospective studies. Second, tNGS detects nucleic acids and cannot distinguish viable pathogens. The targeted panel used had limited coverage, potentially not capturing the full spectrum of the respiratory microbiome. Third, the study coincided with an *M. pneumoniae* outbreak; the observed pathogen epidemiological features and microbial community associations might differ in other epidemic periods. Finally, regarding the confounding effect of antibiotic exposure on the multivariable regression analysis, all samples in this study were collected prior to antibiotic treatment. This minimizes the direct interference of in-hospital therapy on the initial microbial specimen. However, There is a limitation that we were unable to obtain and control for the antibiotic exposure history of most patients prior to hospital admission. Community-acquired antibiotics may have significantly altered the microbial composition of the patients’ upper respiratory tracts. This constitutes a potential, unmeasured important confounding factor that could affect our estimation of the association between microbial features and disease severity. Future prospective studies should systematically collect detailed medication histories prior to admission.

In summary, despite the limitations noted above, throat swab tNGS serves as a powerful, non-invasive tool for mapping the pathogen landscape in pediatric acute respiratory infections. This study establishes *M. pneumoniae* as a key pathogen driving severe pneumonia in the current epidemiological context. Furthermore, it reveals that an altered upper respiratory microbial profile, which is characterized by reduced diversity within a targeted panel and shifted abundance of specific commensals, is associated with pneumonia severity, offering a novel correlative ecological perspective. These findings advocate for a more integrated diagnostic strategy that combines pathogen identification with an assessment of microbial community ecology. While throat swab tNGS is effective for initial screening, cautious interpretation is required to differentiate colonization from true infection, and BALF sampling remains crucial for defining lower respiratory etiology in severe or complex cases.

## Data Availability

The raw data supporting the conclusions of this article will be made available by the authors, without undue reservation.
