# Supplementary material for: Targeted next-generation sequencing analysis of pathogens and microecology in pediatric lower respiratory tract infections identifies risk factors for severe community-acquired pneumonia
Source: Front Cell Infect Microbiol. 2026 Apr 16;16:1796357. doi: 10.3389/fcimb.2026.1796357 (PMC13128607; doi:10.3389/fcimb.2026.1796357)
Supplement: Supplementary file 2 [file Table2.docx]

**Supplementary Methods**

**Construction of Multivariate Regression Analysis Model**

To identify factors independently associated with severe community-acquired pneumonia (SCAP), we constructed a multivariable logistic regression model. The specific steps were as follows:

1. Candidate Variable Screening: Initial candidate variables included all collected clinical features (e.g., age, sex, comorbidities, laboratory parameters) and microbial features detected by tNGS, including the detection status (positive/negative) and relative abundance of key pathogens.
2. Variable Selection and Model Building: First, univariate analysis was performed between all candidate variables and the outcome (SCAP vs. nsCAP). Variables with a P-value < 0.1 in univariate analysis or those deemed clinically significant were retained for inclusion in the multivariable model. We assessed for multicollinearity among the independent variables by calculating the variance inflation factor (VIF). In the final model, the VIF for all variables was < 5, indicating no severe multicollinearity. Continuous variables (e.g., relative abundance) were included in the model on their original scale, and the linearity assumption was assessed. If a clear nonlinear relationship was evident, appropriate transformations (e.g., logarithmic) or treatment as a categorical variable were considered. The results of the final model are presented as adjusted odds ratios (ORs) and p-values. All statistical analyses were performed using R software (version 4.4.1). Logistic regression analysis was primarily conducted using the glm function, and model validation utilized packages such as car and pROC.

**Model Performance and Validation**

The performance of the final model was evaluated and validated as follows: First, its discriminative ability to differentiate SCAP from nsCAP was assessed by calculating the area under the receiver operating characteristic curve (AUC). Second, to evaluate the degree of over-optimism, internal validation of the final model was performed using the Bootstrap resampling method (100 replicates). The optimism-corrected AUC is reported to provide a more accurate estimate of the model's expected performance in future, similar populations.

Based on 100 bootstrap resamples, the model demonstrated moderate overfitting, with a mean optimism of 0.0965 (95% range: 0.0119-0.1594). The original model AUC was 0.785, and the optimism-corrected AUC was 0.688. This indicates that the expected AUC of the model on independent data is approximately 0.688, suggesting it retains a certain degree of predictive ability.

**Supplementary figures**

**Figure S1. Pathogenic spectrum identified through tNGS among 2299 patients. (A) Detection rates of bacteria, viruses, atypical pathogens, and fungi in throat swab samples from 2573 patients. (B) Epidemic trends of M. pneumoniae, B. pertussis, and 20 respiratory viruses during the study period.**

**
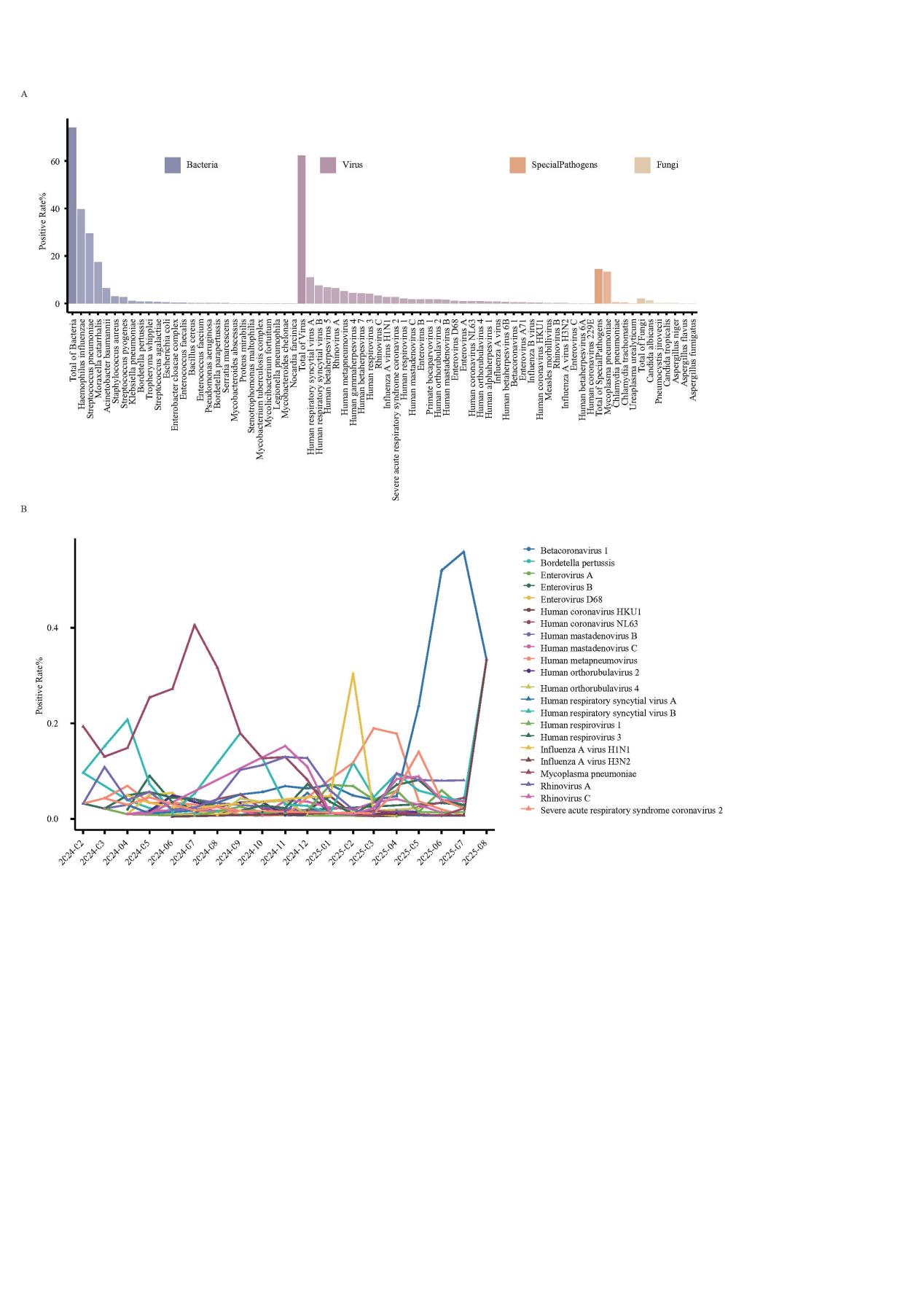
**

**Figure S2. Age-specific pathogen distribution in acute respiratory infections. (A)**​ Detection rates of different pathogen types across age groups. **(B)**​ Detection rates of individual pathogens across age groups (only pathogens with >2 detected cases are shown). **(C)**​ Proportions of single-pathogen-type infections and co-infections of different pathogen types across age groups.


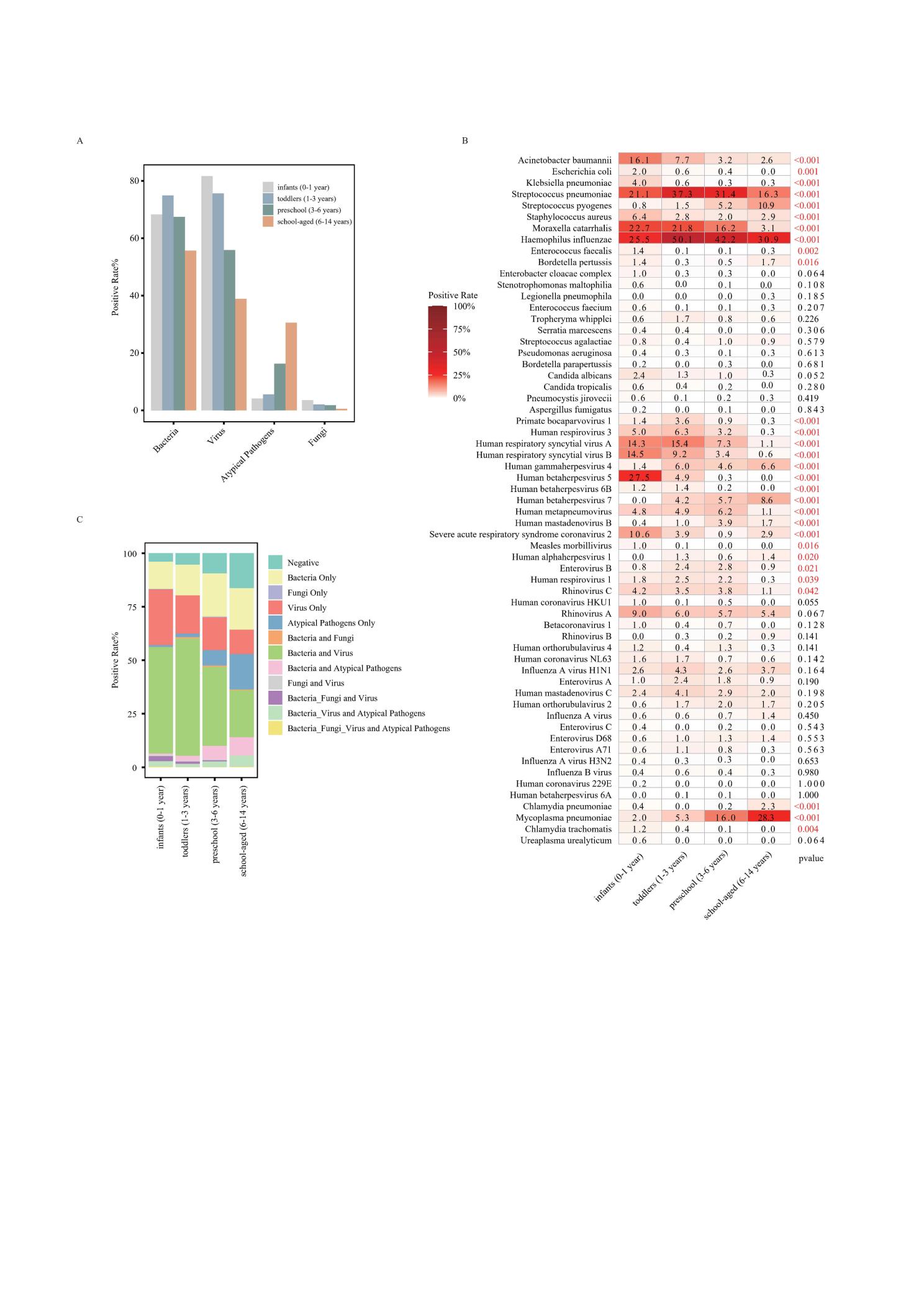


**Figure S3. Co‑infection patterns of 32 clinically significant pathogens in infants (0–1 year) (A), toddlers (1–3 years) (B), preschool children (3–6 years) (C), and school‑aged children (6–14 years) (D).**


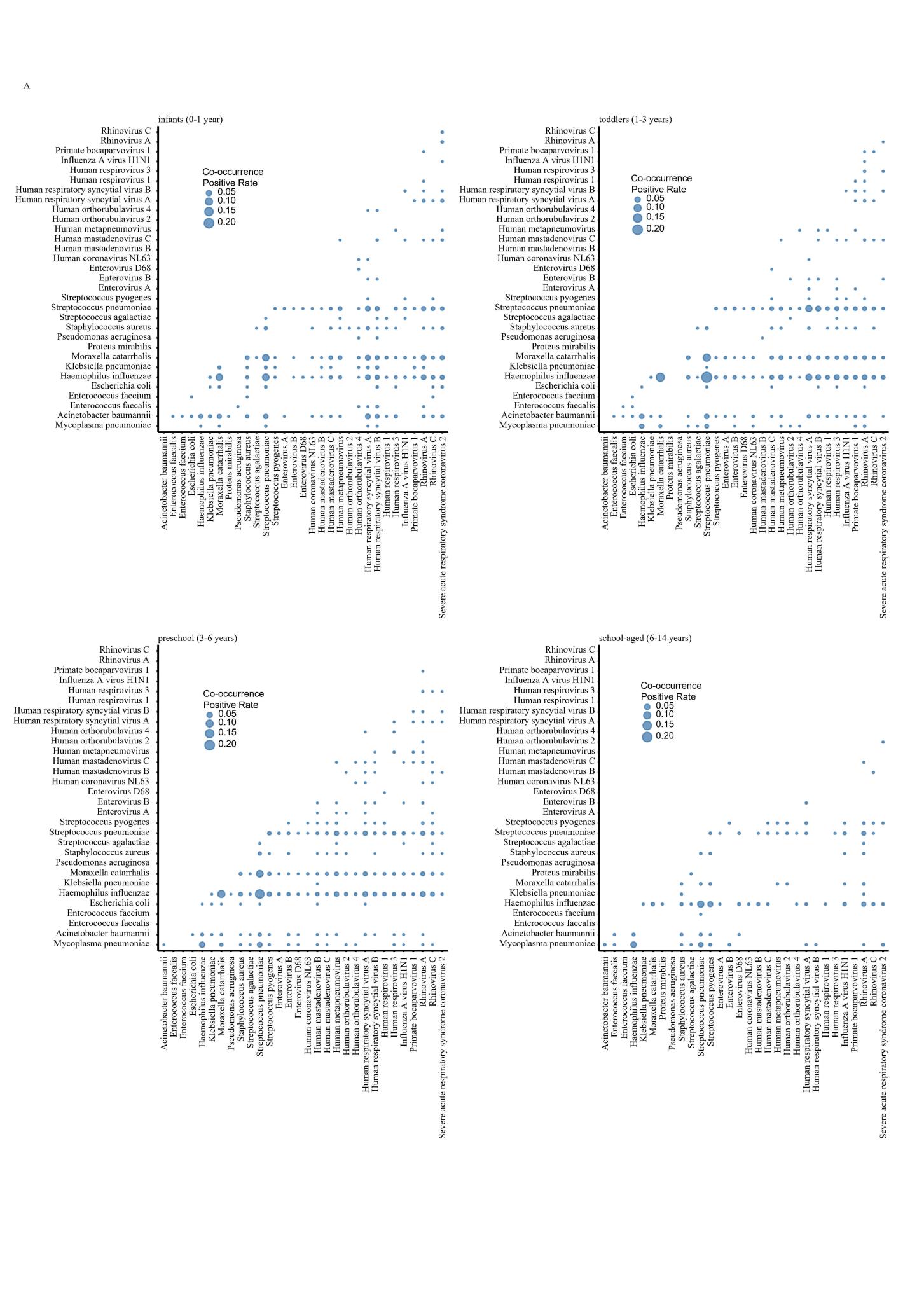


**Figure S4. Distribution of microbial detection rates in severe community-acquired pneumonia (SCAP) and non-severe community-acquired pneumonia (nsCAP) patients, stratified by M. pneumoniae positivity (A) and negativity (B)**

**
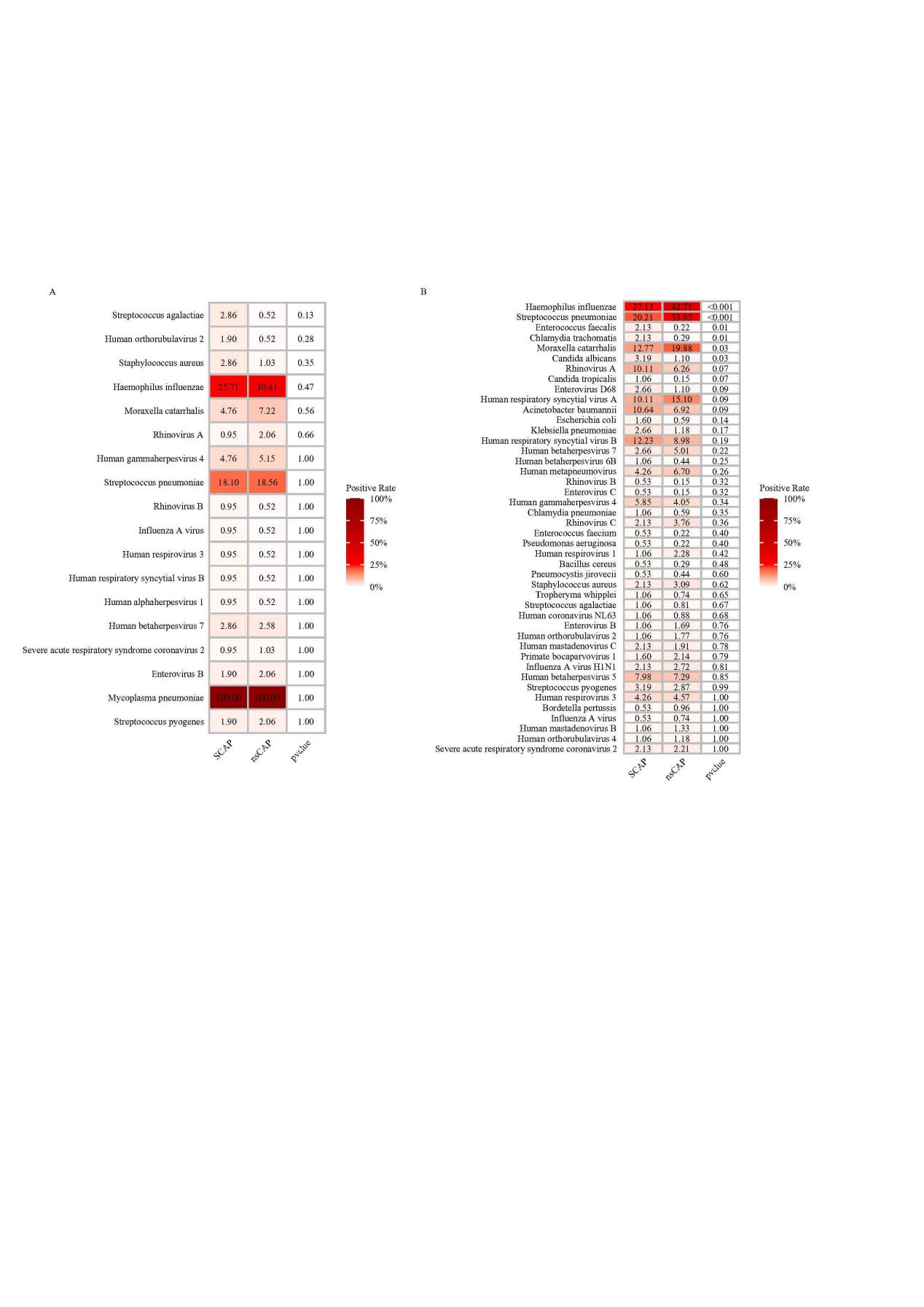
**

**Figure S5. Multivariable-adjusted logistic regression analysis of *M. pneumoniae*-negative patients ( SCAP vs nsCAP) based on detection rate (A) an RA (B).**

**
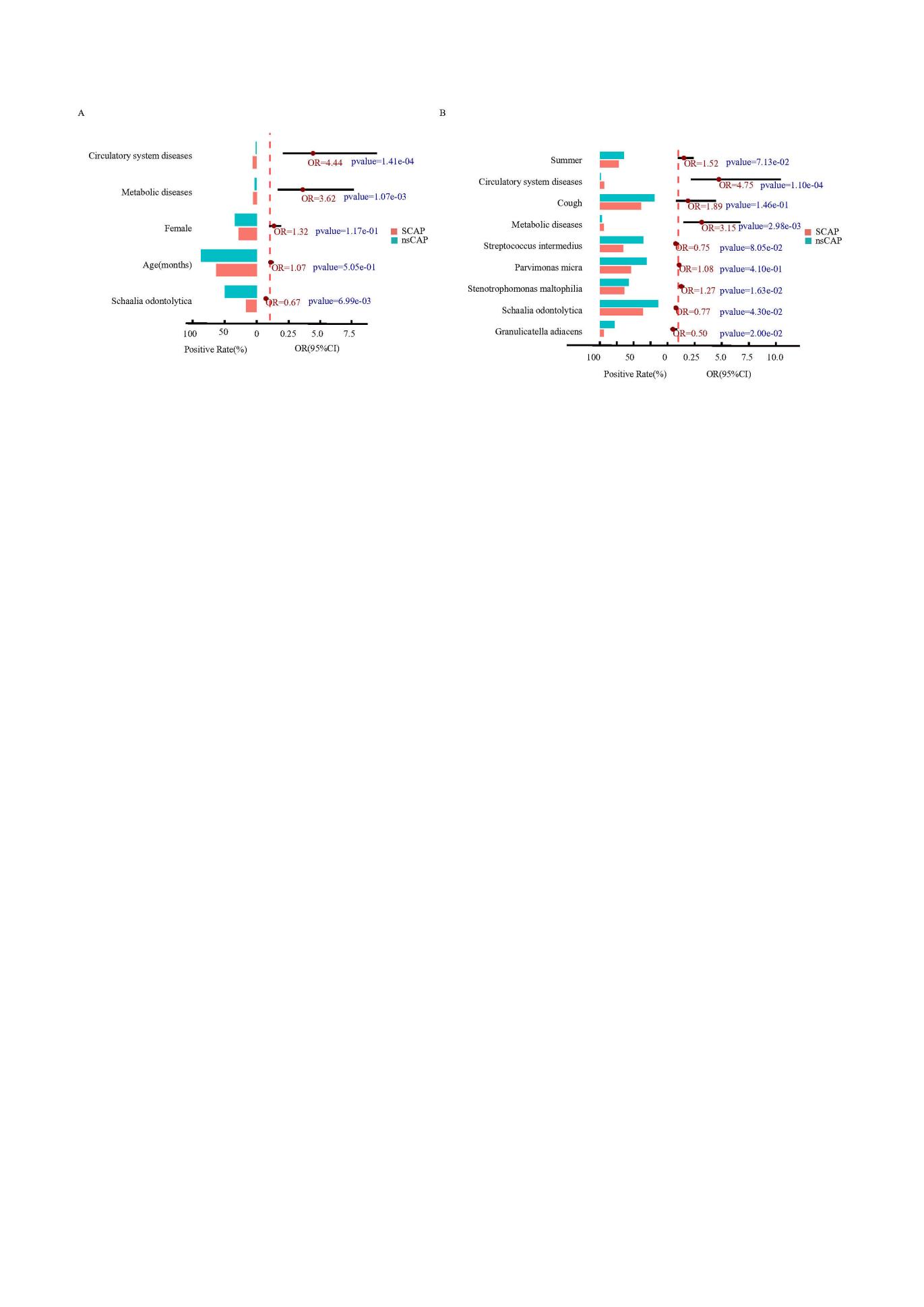
**

**Figure S6. Distribution of Shannon (A) and Simpson (B) indices in SCAP and nsCAP patients with negative *M. pneumoniae* detection.**

**
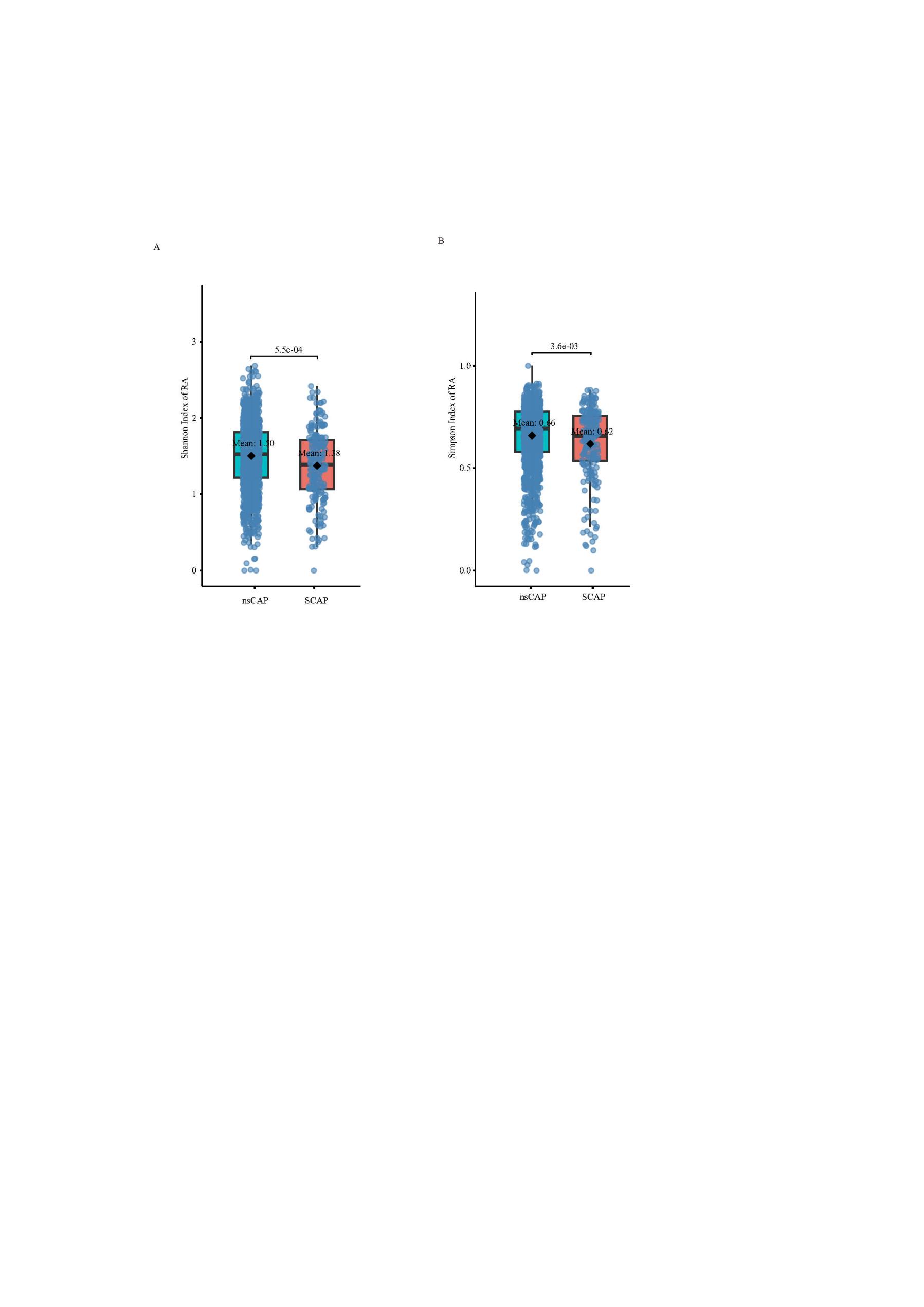
**

**Figure S7. Differences in pathogen detection rates between SCAP and nsCAP patients (complete results)**

**
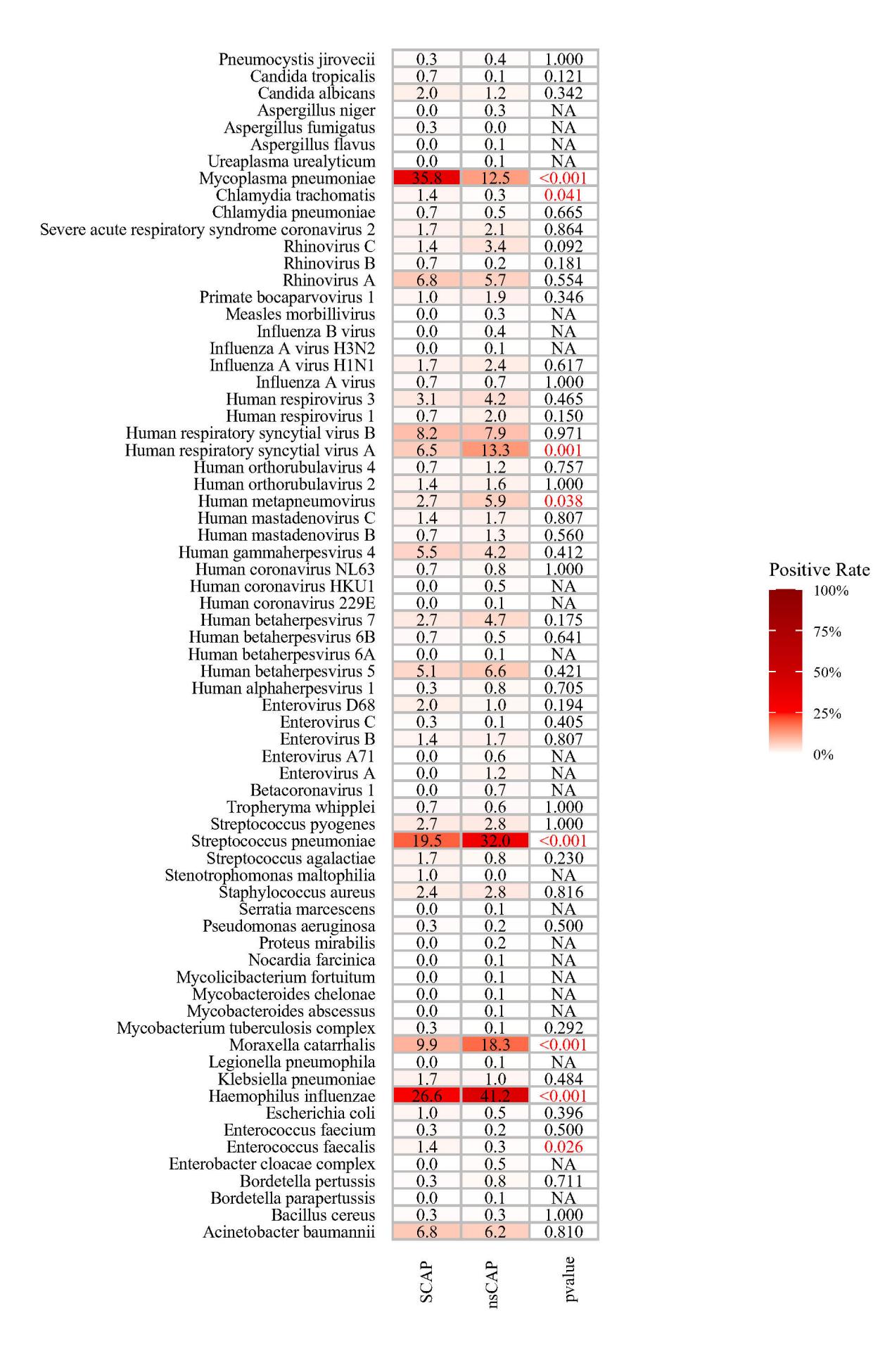
**
